# Extracorporeal membrane oxygenation in immunocompromised patients with acute respiratory failure: A retrospective cohort study

**DOI:** 10.1111/crj.13674

**Published:** 2023-08-27

**Authors:** Ye Tian, Sichao Gu, Xu Huang, Changlong Li, Yi Zhang, Jingen Xia, Yingying Feng, Xin Yu, Ying Cai, Xiaojing Wu, Min Li, Qingyuan Zhan

**Affiliations:** ^1^ National Center for Respiratory Medicine Beijing China; ^2^ State Key Laboratory of Respiratory Health and Multimorbidity Beijing China; ^3^ National Clinical Research Center for Respiratory Diseases Beijing China; ^4^ Institute of Respiratory Medicine Chinese Academy of Medical Sciences Beijing China; ^5^ Department of Pulmonary and Critical Care Medicine, Center of Respiratory Medicine China‐Japan Friendship Hospital Beijing China

**Keywords:** acute respiratory failure, awake ECMO, extracorporeal membrane oxygenation, immunocompromised, nonintubated

## Abstract

**Background:**

The clinical indications of extracorporeal membrane oxygenation (ECMO) in immunosuppressed patients are not clear. This study aimed to analyse the effectiveness of ECMO and to identify the risk factors for the mortality of ECMO in immunocompromised patients with acute respiratory failure.

**Methods:**

This retrospective, cohort study included 46 confirmed immunocompromised patients with acute hypoxemic respiratory failure treated with ECMO between July 2014 and August 2020. The clinical features and outcomes of the survival group and the non‐survival group were statistically analysed.

**Results:**

The mean age of the enrolled patients was 60.0 (50.0, 66.0) years; male patients accounted for 60.9% of patients, and the mean CD4 level was 213 cells/μL (150.3, 325.3). The hospital mortality rate of the cohort was 67.4% (31/46 patients). Patients in the survival group showed a higher rate of receiving awake ECMO (11/15 vs. 4/31; *p* = 0.006), a lower rate of acute kidney injury (AKI) receiving continuous renal replacement therapy (CRRT) (1/15 vs. 12/31; *p* = 0.035), fewer platelet transfusion units (0/15 vs. 2/31 units; *p* = 0.039) and a lower rate of ventilator‐associated pneumonia (2/15 vs. 19/31; *p* = 0.006). In a multivariate Cox regression analysis model, intubated ECMO (hazard ratio = 1.77, 95% confidence interval: 1.34–2.32, *p* < 0.001) and AKI requiring CRRT (1.37, 95% confidence interval: 1.14–1.61, *p* = 0.003) were identified as independent risk factors for mortality.

**Conclusions:**

In‐hospital mortality has remained high in ECMO‐treated immunocompromised patients with acute respiratory failure. Intubated ECMO and AKI receiving CRRT during ECMO treatment may predict ECMO failure in immunocompromised patients with ARF. A primarily awake ECMO strategy seems feasible in some selected immunocompromised patients.

## Graphical abstract

ECMO support for immunosuppressed patients remains controversial and challenging. This study provides detailed information on the clinical and laboratory features of these critical patients. Our findings provide insight into the in‐hospital mortality has remained high in such patients. Interesting things are, a primarily awake ECMO strategy seems feasible in some selected patients and acute kidney injury requiring CRRT during ECMO treatment may predict ECMO failure in such patients.

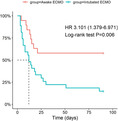

**Abbreviations**
AKI: acute kidney injury
ARDS: acute respiratory distress syndrome
ARF: acute respiratory failure
CI: confidence interval
CRRT: continuous renal replacement therapy
CS: corticosteroids
ECMO: extracorporeal membrane oxygenation
IMV: invasive mechanical ventilation
IS: immunosuppressant
PaCO_2_
: partial pressures of carbon dioxide
PaO_2_
: partial pressures of oxygen
PFR: PaO_2_/FiO_2_ ratio
VAP: ventilator‐associated pneumonia

## 1 INTRODUCTION

The worldwide use of extracorporeal membrane oxygenation (ECMO) is rapidly increasing for severe acute respiratory failure (ARF) patients.^1^ However, the negative impacts of immunosuppression as a relative exclusion criterion of ECMO therapy on the survival of such patients have been constantly emphasized.^2^, ^3^, ^4^ Indeed, 19% to 31% of ECMO‐supported patients with ARF in recently published cohorts were immunocompromised.^3^ Nevertheless, the in‐hospital mortality rate for this population remains high in the past decade, at 50% to 100%.^5^, ^6^, ^7^, ^8^, ^9^, ^10^ The high fatality rate is mainly due to complications related to mechanical ventilation or ECMO, such as ventilator‐associated pneumonia (VAP), lung injury, septic shock, haemorrhage and thrombotic events.^11^ Current data on ECMO support in this specific population were mainly described in some single‐centre cohort studies with a small number of patients, and the risk factors for mortality and prognosis have not yet been thoroughly examined.^6^ Therefore, the objectives of this study were to explore the clinical indications for ECMO in immunocompromised patients with ARF by analysing the outcomes and predicted risk factors for mortality in those patients.

## 2 PATIENTS AND METHODS

### 2.1 Study design

We retrospectively studied the clinical courses of all immunocompromised recipients treated with ECMO for ARF in the medical intensive care unit of our hospital between July 2014 and August 2020.

### 2.2 Inclusion criteria and exclusion criteria

All immunocompromised recipients (18 years old or older) treated with ECMO for ARF in our centre were enrolled in the study. We defined ‘immunocompromised’ patients as having at least one of the following conditions: (1) immunosuppression, which was defined as patients with primary immune deficiency, neoplastic disease, immunosuppressive drugs receiving long‐term (>3 months) or high‐dose (>0.5 mg/kg/day) corticosteroids (CS) or other immunosuppressant (IS) drugs and/or (2) active hematologic malignancy and neoplasm (i.e., still requires treatment, has not been resected or is metastatic).^12^

**FIGURE 1:**
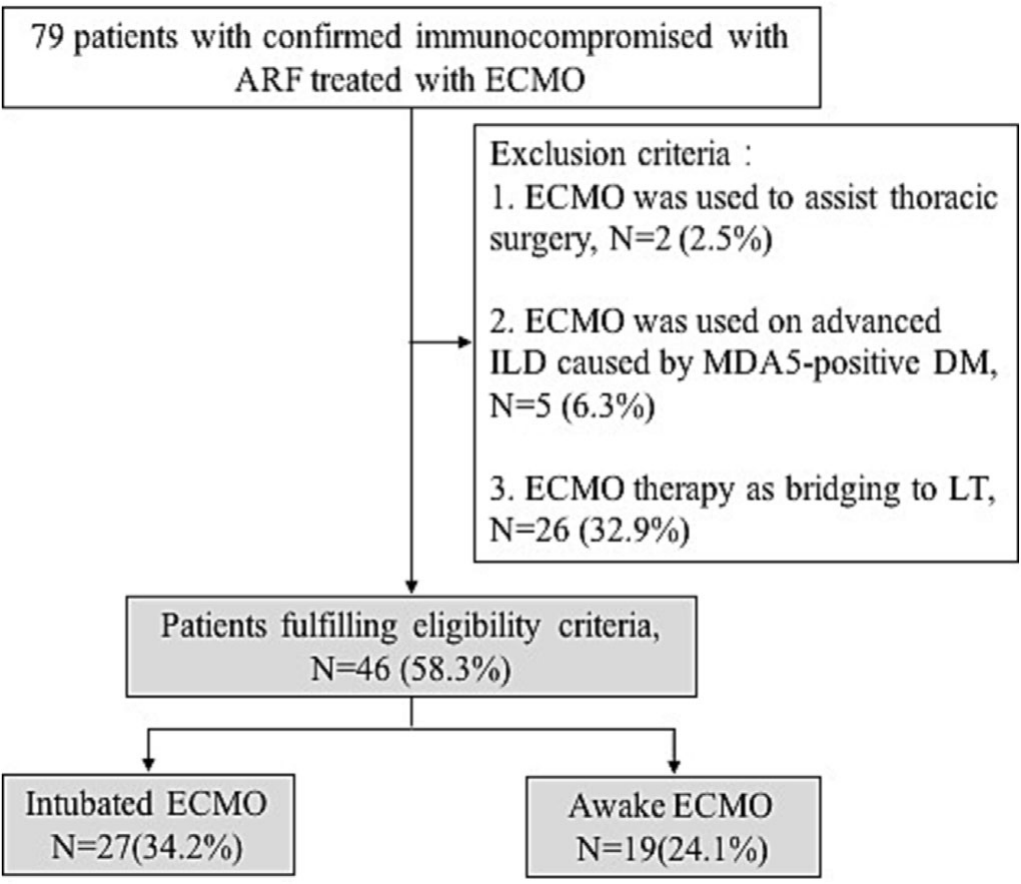
Flow chart of the study. ARF, acute respiratory failure; ECMO, extracorporeal membrane oxygenation; ILD, interstitial lung disease; MDA5, melanoma differentiation‐associated protein 5; DM, dermatomyositis, LT, lung transplantation.

We excluded patients who underwent ECMO therapy as bridging to lung transplantation or thoracic surgery and would not benefit from ECMO, such as patients with advanced interstitial lung disease caused by MDA5‐positive dermatomyositis, which is known to have a poor prognosis. The flow chart of the study is shown in Figure 1.

### 2.3 ECMO implementation

ECMO was initiated in the event that the PaO_2_/FiO_2_ ratio (PFR) was less than 80 mmHg over 6 h (FiO_2_ > 0.9). ECMO weaning was also performed in accordance with the Extracorporeal Life Support Organization guidelines.^13^ All patients were treated with the Rotaflow Console ECMO machine (Maquet Cardiopulmonary Gmbh, Kehler Str.31, 76 437 Rastatt, Germany). Ultrasound‐guided percutaneous cannulation was performed by inserting a 21F or 23F drainage cannula (Maquet) into a femoral vein and a 15F or 17F cannula (Maquet) into a jugular vein using the Seldinger technique. Patients with a thrombocyte count of less than 50 × 10^9^/L received two thrombocyte concentrates prior to cannulation. The blood flow was set to reach adequate systemic oxygenation; the sweep gas flow was set to reach normal PaCO_2_. Anticoagulation was initially led with continuous intravenous infusion of unfractionated heparin to maintain an activated partial thromboplastin time of 50–70 s in all patients.

### 2.4 Definition

We defined intubated ECMO patients as those who underwent invasive mechanical ventilation (IMV) before ECMO application after the event of severe respiratory failure and awake ECMO as those who underwent ECMO therapy in awake, nonintubated, spontaneously breathing patients as an alternative to IMV.

We defined patients who had a high risk of failure and would not benefit from awake ECMO based on clinical assessment such as those with (1) age>75 years old, (2) impaired mental status (Glasgow coma score <9), (3) haemodynamic disorder (requiring vasopressor therapy), (4) high risk of aspiration (inability to protect airway) and (5) excessive airway secretions or unclear cause of ARF.^14^, ^15^ These decisions to use awake ECMO were made by a multidisciplinary team including intensivists and pulmonologists.

Bleeding events were defined as the requirement of four or more units of red blood cells due to obvious bleeding events and noted as if an interventional procedure was required, as well as in cases of fatal outcomes such as intracerebral haemorrhage.

### 2.5 Data collection

The time point immediately before the start of awake ECMO treatment was referred to as the baseline. Baseline data were recorded for the time immediately before initiation of ECMO therapy. Age, sex, cause of ARF, body mass index, types of immunosuppression and intention of underlying lung status were recorded. The sequential organ failure assessment score and APACHE II score were calculated at baseline to grade the severity of illness. The cause of ARF was determined, and arterial blood gas parameters such as pH, partial pressures of oxygen (PaO_2_), carbon dioxide (PaCO_2_) and lactate; PFR; laboratory findings such as white blood cell counts, platelet counts and haemoglobin; and CD4 levels were recorded at baseline. Furthermore, treatment with steroids and the need for continuous renal replacement therapy (CRRT) with acute kidney injury (AKI) patients were determined (Table S1).

### 2.6 Statistical analyses

Continuous variables are expressed as the mean ± SD or median, whereas categorical values are expressed using relative frequencies and proportions. Data were compared between survivors and nonsurvivors using Fisher's exact test for dichotomous variables and the Mann–Whitney *U* test or independent *t*‐test for continuous variables. Univariate and multivariate analyses were carried out to define independent factors strongly associated with mortality. Cox proportional hazard models were constructed to estimate the hazard ratio and 95% confidence interval (CI). The survival nomogram was developed on the basis of a multivariable Cox model. Kaplan–Meier statistics were used to estimate survival rates with statistical significance assessed by log‐rank analysis. Differences were considered statistically significant when the p value was less than 0.05. Statistical tests were performed using R version 3.6.3 (2020‐02‐29) ‐ ‘Holding the Windsock’ Copyright (C) 2020 The R Foundation for Statistical Computing.

## 3 RESULTS

A total of 79 confirmed immunocompromised patients were admitted, and all were treated with venovenous ECMO (VV‐ECMO) in our centre. We excluded 2 patients who underwent ECMO to assist thoracic surgery, 5 patients who underwent ECMO because of advanced interstitial lung disease caused by MDA5‐positive dermatomyositis, and 26 who received ECMO therapy as a bridge to lung transplantation (Figure 1). Finally, a total of 46 patients fulfilling our eligibility criteria were included in the cohort. The mean age was 60.0 (50.0, 66.0) years, male patients accounted for 60.9% (28 patients) of the cohort, and the mean body mass index was 24.2 ± 3.0 kg/m^2^. Types of immunosuppression were long‐term/high‐dose steroids or other immunosuppressant drugs (91.2%), active hematologic malignancy (4.4%), and active solid organ malignancy (4.4%), and the mean CD4 level was 213 cell/μL (150.3, 325.3). The interval time between the diagnosis of immunosuppression and ECMO support days was 60.0 (20.3119.0) days. The sequential organ failure assessment and APACHE II scores were 6.0 (5.0, 8.0) and 20.0 (16.3, 24.0), respectively, after admission. The PFR and respiratory rate were 66.5 ± 14.4 and 32.4 ± 7.3 before ECMO therapy, respectively. Nineteen (41.3%) and 27 (58.7%) patients underwent ‘awake’ VV‐ECMO and intubated VV‐ECMO, respectively. During ECMO therapy, 32 (69.6%) patients received vasopressors, 13 (28.3%) AKI patients received CRRT therapy, and 44 (95.7%) patients received continuous intravenous infusion of unfractionated heparin. The incidences of bleeding events, pulmonary barotrauma, VAP, ECMO related cannula infection and cardiogenic shock were 67.4%, 30.4%, 45.7%, 10.9% and 28.3%, respectively (Table 1).

**TABLE 1:** ICU and extracorporeal membrane oxygenation‐related characteristics and outcome.

Variables	Total (*n* = 46)	Survivor (*n* = 15)	Nonsurvivor (*n* = 31)	*p*
Characteristics at ICU admission				
Gender, *n* (%)				0.090
Female	18 (39.1)	9 (60.0)	9 (29.0)	
Male	28 (60.9)	6 (40.0)	22 (71.0)	
Age, median (IQR)	60.0 (50.0, 66.0)	59.0 (50.5, 64.0)	60.0 (48.0, 68.0)	0.519
BMI, mean ± SD	24.2 ± 3.0	23.8 ± 2.7	24.5 ± 3.1	0.462
Type of immunosuppression, *n* (%)				1
Long‐term/high‐dose steroids or other immunosuppressant drugs	42 (91.2)	14 (93.3)	28 (90.3)	
Active hematologic malignancy	2 (4.4)	0 (0)	2 (6.5)	
Active solid organ malignancy	2 (4.4)	1 (6.7)	1 (3.2)	
Interval time between the diagnosis of immunosuppression and ECMO support days, median (IQR)	60.0 (20.3119.0)	55.0 (20.5,85.0)	60.0 (20.5120.0)	0.590
SOFA, median (IQR)	6.0 (5.0, 8.0)	7.0 (5.0, 7.5)	6.0 (5.0, 8.0)	0.643
Apache II, median (IQR)	20.0 (16.3, 24.0)	19.0 (15.5, 23.0)	21.0 (17.0, 24.5)	0.459
Characteristics at ECMO baseline				
Pre ECMO PaO_2_/FiO_2_ ratio mmHg, mean ± SD	66.5 ± 14.4	67.2 ± 18.6	66.2 ± 12.2	0.847
Pre ECMO pH, median (IQR)	7.39 (7.29, 7.44)	7.41 (7.36, 7.47)	7.38 (7.28, 7.43)	0.223
Pre ECMO PaCO_2_ mmHg, median (IQR)	45.9 (36.4, 55.9)	41.0 (35.6, 51.6)	47 (36.8, 56.3)	0.439
Lactate mmol/L, median (IQR)	1.7 (1.4, 2.2)	1.8 (1.5, 3.1)	1.5 (1.4, 2.2)	0.397
CD_4_ level cell/μL, median (IQR)	213 (150.3, 325.3)	306 (168.0, 361.5)	204 (130, 315)	0.261
Leucocytes count (×10^9^/L), mean ± SD	13.4 ± 7.0	15.3 ± 8.8	12.5 ± 6.0	0.266
Neutrophil count (×10^9^/L), median (IQR)	10.9 (7.9, 16.8)	12.7 (8.5, 18.1)	10.4 (7.3, 16.5)	0.412
Lymphocyte count (×10^9^/L), median (IQR)	0.6 (0.3, 0.7)	0.6 (0.4, 0.9)	0.5 (0.3, 0.7)	0.052
Platelets (×10^9^/L), median (IQR)	144.5 (107, 220.5)	156 (101.5, 240)	138 (113, 209)	0.725
Haemoglobin g/L, mean ± SD	107.2 ± 22.3	112.5 ± 22.6	104.7 ± 22.1	0.281
Lactate dehydrogenase IU/L, median (IQR)	521.5 (387.5, 743.5)	523 (370, 680.5)	520 (399, 763.5)	0.806
C‐reactive protein mg/dL, median (IQR)	8.1 (5.2, 15.2)	8.5 (4.9, 19.2)	7.4 (5.2, 13.9)	0.752
Pre ECMO RR, mean ± SD	32.4 ± 7.3	33.8 ± 5.0	30.5 ± 6.7	0.118
Type of ECMO, *n* (%)				0.006
Awake ECMO	19 (41.3)	11 (73.3)	8 (25.8)	
Intubated ECMO	27 (58.7)	4 (26.7)	23 (74.2)	
Characteristics during ECMO				
Post ECMO RR, mean ± SD	23.0 ± 6.3	21.3 ± 5.1	23.9 ± 6.7	0.164
Vasopressors, *n* (%)	32 (69.6)	8 (53.3)	24 (77.4)	0.17
CRRT, *n* (%)	13 (28.3)	1 (6.7)	12 (38.7)	0.035
Anticoagulant therapy, *n* (%)	44 (95.7)	15 (100)	29 (93.6)	1
Bleeding events, *n* (%)	31 (67.4)	9 (60)	22 (71.0)	0.514
RBC transfusion units, median (IQR)	4.5 (2, 8)	4 (2, 8)	5 (2, 8)	0.897
Platelet transfusion units, median (IQR)	1 (0, 2)	0 (0, 1)	2 (0, 2.5)	0.039
Steroids during ECMO, *n* (%)	40 (87.0)	14 (93.3)	26 (83.9)	0.647
Mediastinal emphesema/pneumothorax, *n* (%)	14 (30.4)	6 (40)	8 (25.8)	0.495
VAP, *n* (%)	21 (45.7)	2 (13.3)	19 (61.3)	0.006
ECMO related cannula infection, *n* (%)	5 (10.9)	0 (0)	5 (16.1)	0.253
None, *n* (%)	13 (28.3)	1 (6.7)	12 (38.7)	0.056
Outcomes				
Weaning of ECMO, *n* (%)	17 (37.0)	15 (100)	2 (6.5)	< 0.001
Refusing continuous ECMO treatment, *n* (%)	14 (30.4)	0 (0)	14 (45.2)	0.005
Duration of ECMO therapy, median (IQR)	12.5 (6.3, 18.8)	15 (9, 21)	12 (5.5, 17.5)	0.227
ICU length of stay, median (IQR)	18.0 (12, 27)	25 (19.0, 37.5)	14 (10.5, 23.0)	0.008
Hospital length of stay, median (IQR)	24 (15, 37)	35 (25, 50)	21 (13.5, 33)	0.03
ICU and hospital survival	15 (32.6)			

Abbreviations: BMI, body mass index; CRRT, continuous renal replacement therapy; ECMO, extracorporeal membrane oxygenation; ICU, intensive care unit; IQR, interquartile range; RBC, red blood cell; RR, respiratory rate; SOFA, sequential organ failure assessment; VAP, ventilator associated pneumonia.

### 3.1 Clinical outcomes

**FIGURE 2:**
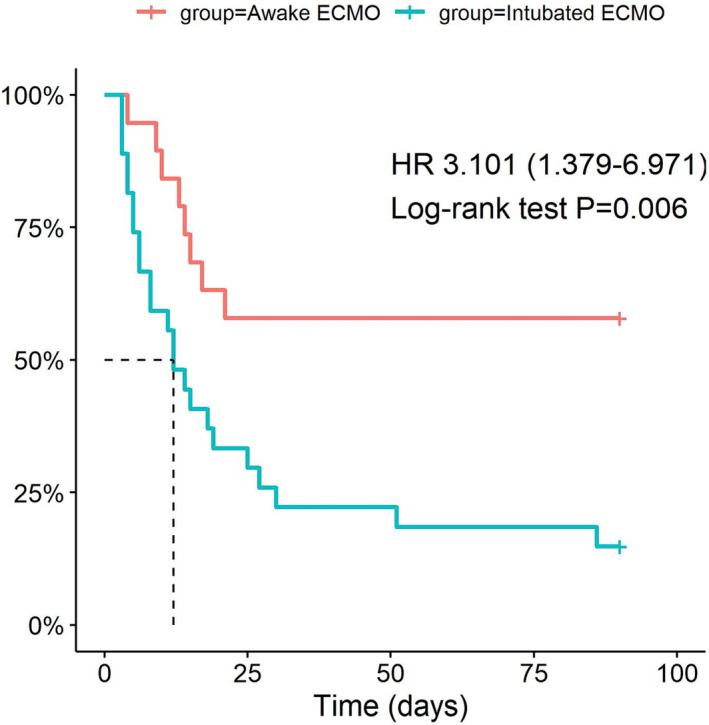
Survival in awake versus intubated ECMO immunocompromised patients with severe ARF. Kaplan–Meier graphs showing the survival course from application of ECMO to discharge in awake (*n* = 19) and intubated (*n* = 27) with immunocompromised associated ARF (*p* = 0.006).

The overall hospital mortality rate of the immunocompromised patient cohort was 67.4% (31/46 patients). However, 41.3% (19/46) of patients avoided endotracheal intubation in the early phase of disease (applied awake ECMO), and 11 of them lived and were discharged from the hospital (Table 1). The median length of ECMO therapy was 12.5 days (6.3, 18.8 days), and the median length of hospital stay was 24 days (15, 37 days). The awake ECMO group had a distinct prognosis compared with the primarily intubated ECMO group according to Kaplan–Meier curves for survival outcomes (*p* = 0.006, Figure 2).

### 3.2 Characteristics of survivors and nonsurvivors

In the cohort, 15 patients (32.6%) lived and were discharged from the hospital. Patients in the survival group showed a higher rate of receiving awake ECMO (11/15 vs. 4/31; *p* = 0.006), a lower rate of AKI receiving CRRT (1/15 vs. 12/31; *p* = 0.035), fewer platelet transfusion units (0/15 vs. 2/31 units; *p* = 0.039) and a lower rate of ventilator‐associated pneumonia (2/15 vs. 19/31; *p* = 0.006) (Table 1). As shown in Table 1, there were four notable differences in the characteristics of survivors and nonsurvivors. These parameters with *p* values less than 0.05 were then included in a univariate Cox model (Table 2). Univariate analysis indicated that two factors (type of ECMO and CRRT during ECMO therapy) were associated with the prognosis of the immunocompromised patients with ARF (*p* < 0.05). The two significant factors with high HRs in the univariable analysis were entered into a multivariable analysis on the basis of the Cox regression. Multivariable analysis indicated that intubated ECMO (hazard ratio = 1.77, 95% CI: 1.34–2.32, *p* < 0.001) and CRRT (1.37, 95% CI: 1.14–1.61, *p* = 0.003) were identified as independent risk factors for mortality (Table 2).

**TABLE 2:** Univariate and multivariate cox regression analyses of variables significantly associated with mortality.

Variables	Univariate analysis			Multivariate analysis		
	βcoefficient	HR (95% CI)	*p*	βcoefficient	HR (95% CI)	*p*
Type of ECMO	0.957	2.60 (1.16–5.87)	0.021	0.910	2.48 (1.10–5.63)	0.029
CRRT	1.316	3.73 (1.74–7.99)	<0.001	1.298	3.66 (1.69–7.94)	0.001
Platelet transfusion units	0.066	1.07 (0.95–1.20)	0.266			
VAP	0.595	1.81 (0.87–3.78)	0.112			

Abbreviations: CI, confidence interval; CRRT, continuous renal replacement therapy; ECMO, extracorporeal membrane oxygenation; HR, hazard ratio; VAP, ventilator associated pneumonia.

## 4 DISCUSSION

This report describes a cohort of 46 immunocompromised patients treated with ECMO for severe ARF. The following are the main results: (1) In‐hospital mortality has remained high in ECMO‐treated immunocompromised patients with ARF, and (2) intubated ECMO therapy and CRRT during ECMO therapy were independent risk factors for the mortality of immunocompromised patients with ARF.

ECMO has been increasingly used on severe ARF patients in recent years. Generally, the ideal candidate for ECMO should be one with known reversible disease, less organ failure, minimal co‐morbidity, nervous system integrity and minimal risk of bleeding.^13^ In practice, few of the patients cared for in modern intensive care units (ICUs) fit this description. The state of the patient's immune system is an important consideration in determining whether the patient is suitable for ECMO support. However, its use has infrequently been reported according to the high mortality in immunocompromised patients, especially when mechanical ventilation is required or ECMO‐related complications occur.^2^ A recent subanalysis of the LUNG SAFE study highlights the independent association of active cancer, hematologic malignancy, or immunodeficiency with increased in‐hospital mortality in acute respiratory distress syndrome (ARDS) patients.^4^ In addition, Extracorporeal Life Support Organization guidelines still consider ‘immunosuppression (absolute neutrophil count <400/mm^3^)’ a relative contraindication for several reasons: (1) lymphocyte reduction or leukopenia from chemotherapy confers a risk of subsequent infection, (2) multiple organ failure usually occurs before ECMO initiation, (3) patients with coagulopathy are at a higher risk for haemorrhagic complications and (4) uncertainty of patient's long‐term prognosis with respect to their underlying disease.^13^ These factors all lead to difficulties in ECMO treatment and extremely high fatality rates in immunosuppressed patients with ARF.

In this cohort, the hospital mortality rate was 67.4%, which was similar to the results of other studies.^7^, ^16^ Schmidt described the largest cohort of immunocompromised patients treated with ECMO for moderate or severe ARDS (i.e., 203 patients from seven countries over 8 years). In his cohort, the 6‐month survival rates were 40%, 37%, 26%, 24% and 20% in the solid‐organ transplant, long‐term or high‐dose CS or IS, AIDS, haematological malignancies and solid tumour groups, respectively. Compared with other immunocompromised patients, patients with haematological malignancies had a significantly worse outcome. ECMO‐related major bleeding, cannula infection and ventilator‐associated pneumonia were frequent (36%, 10% and 50%, respectively) in the study.^5^ In our study, the survival rates were 33.3% (14/42), 0% (0/2) and 50% (1/2) in the long‐term or high‐dose CS or IS, haematological malignancy and solid tumour groups, respectively. The incidences of bleeding events, pulmonary barotrauma, VAP, ECMO related cannula infection and cardiogenic shock were 67.4%, 30.4%, 45.7%, 10.9% and 28.3%, respectively.

We suggest that support for the use of ECMO in immunosuppression may stem from the notion that patients with stable primary disease, less organ dysfunction, and ECMO‐related complications can be avoided. In terms of ECMO complications, ECMO‐related infection, bleeding and thrombosis events are the most concerning. Awake ECMO may be an effective way to prevent VAP in our study. The prevention of bleeding events depends not only on the patients' own coagulation function but also on the monitoring of coagulation indicators and adequate anticoagulation therapy in daily work.

In addition, immunocompromised patients have a high fatality rate with conventional IMV, which is associated with an increased incidence of VAP and barotrauma.^10^ Awake nonintubated ECMO may help prevent such complications.^17^ Eleven patients in our study successfully avoided intubation and survived, and the incidence of VAP and barotrauma was 45.7% and 30.4%, respectively. The advantages of awake ECMO might be as follows: (1) Maintaining spontaneous breathing can lead to optimal ventilation–perfusion matching, reduce the incidence of atelectasis, maintain diaphragmatic contraction, avoid diaphragm paralysis, and reduce the incidence of ventilator/intubation‐associated pneumonia through the maintenance of natural barrier defenses against bacteria.^1^, ^18^ (2) By reducing the use of sedatives in the ICU, which is associated with prolonged ICU/hospital stay and mortality, awake patients can actively collaborate with rehabilitators to perform physical rehabilitation to reduce the incidence of neuromuscular disorders.^19^, ^20^ In recent years, some centres have started to use ECMO as a first line of treatment, that is, as an alternative to IMV in awake, nonintubated, spontaneously breathing patients, such as those undergoing bridging to lung transplantation^21^, ^22^ or suffering from end‐stage chronic obstructive pulmonary disease^23^, ^24^ or ARDS,^25^, ^26^ but the use of awake ECMO in immunocompromised patients has so far been reported only as single cases or in small series thus far.^27^, ^28^, ^29^ A recent study suggests that the use of awake ECMO in immunocompromised patients with pneumocystis *jirovecii* pneumonia and severe ARDS appears to be a strategy for improving prognosis.^30^ Klaus Stahl et al. summarized 18 immunocompromised patients receiving awake ECMO for ARDS and suggested that the awake ECMO strategy may be an appropriate choice for severe ARDS patients with compromised immune function.^31^ However, during awake ECMO therapy, spontaneous breathing efforts have also been demonstrated as self‐inflicted lung injury.^25^ Lung damage might therefore also derive from spontaneous hyperventilation (spontaneous ventilation‐induced lung injury), and high effort of the strenuous respiratory muscle can lead to high oxygen (O_2_) consumption and carbon dioxide (CO_2_) production.^32^ In our retrospective analysis, we cannot solve this important concern, but we did observe that respiratory rates after awake ECMO therapy significantly declined in the survivor group (Table 2), indicating clinical relief of patients' respiratory distress. More research on the influence of self‐inflicted lung injury with awake ECMO therapy is required.

Moreover, 13 AKI patients received CRRT in our study, and there was a statistically significant difference between the survival and nonsurvival groups. All 13 patients underwent the introduction of a hemofiltration filter into the ECMO circuit, which is the most widely used method of CRRT and has the advantage of being simple, inexpensive and less traumatic. CRRT is commonly used in ICUs to provide renal replacement and fluid management in critically ill patients with acute cardiac and/or pulmonary dysfunction who are at high risk for AKI and fluid overload. Studies have shown that over 30% of patients receiving ECMO have AKI, and the prognosis is worse than in those without renal failure.^33^ Therefore, it is often necessary to combine CRRT with ECMO in clinical practice. Schmidt et al. recently found that 60% of 172 adult patients in the medical community were average, which combined with the medical community was generally an independent predictor of 90‐day mortality.^34^ Studies found that in‐hospital mortality in ECMO combined with CRRT was significantly higher than that in ECMO alone (OR 5.89, 95% CI: 4.38–7.92, *p* < 0.00001).^35^, ^36^, ^37^ A systematic review of the combination of ECMO and CRRT reported the in‐hospital mortality of 13 studies, with a statistically significant increase in the risk of mortality in ECMO combined with CRRT patients compared with patients receiving ECMO only (OR 5.89, 95% CI 4.38 to 7.92, *p* < 0.00001).^38^ Thus, as a component of multiple organ dysfunction syndrome, the presence of AKI itself rather than the requirement for CRRT is an independent risk factor for mortality in critically ill patients undergoing ECMO.

This study has the following limitations. First, this study is a retrospective study that included a small number of patients in a single‐centre setting. In addition, the distribution of immunosuppressed types was uneven, and the limitation of patient selection was represented as there were a small number of patients who had haematological malignancies and solid tumours. There is obvious selection bias that ECMO may be often used for those receiving long‐term immunosuppressive drugs or high‐dose steroid patients, which might have influenced the outcomes. Third, we did not provide extended data on a strategy for ECMO and ventilator settings, which is a common limitation of most observational and interventional ECMO trials due to the individualized treatment of ECMO and avoidance of endotracheal intubation in some patients. Fourth, the sample size was too small for a matched pairs analysis between the survival group and the nonsurvival group or even between the awake ECMO and intubated ECMO of immunocompromised patients, thus confirming that awake ECMO can actually reduce the mortality of immunosuppressed patients, although there are still many disadvantages of awake ECMO, and it has not been widely used. In the future, prospective studies might be designed to expand the sample size to obtain more reliable evidence.

## 5 CONCLUSIONS

In‐hospital mortality has remained high in ECMO‐treated immunocompromised patients with ARF. A primarily awake ECMO strategy seems feasible in some selected immunocompromised patients. AKI receiving CRRT during ECMO treatment may predict ECMO failure in immunocompromised patients with ARF.

## AUTHOR CONTRIBUTIONS

Qingyuan Zhan conceived of and designed the study, had full access to all of the data in the study, and takes responsibility for the integrity of the data and accuracy of the data analysis. Ye Tian drafted the paper. Min Li, Xu Huang, Changlong Li, Sichao Gu and Yi Zhang. Min Li, Ye Tian, Jingen Xia, Yingying Feng, Xin Yu, Ying Cai and Xiaojing Wu cared for the critical patients and performed the analysis, and all authors critically revised the manuscript for important intellectual content and gave final approval for the version to be published. All authors agree to be accountable for all aspects of the work in ensuring that questions related to the accuracy or integrity of any part of the work are appropriately investigated and resolved.

## CONFLICT OF INTEREST STATEMENT

We declare that there are no competing interests.

## ETHICS STATEMENT

The study was approved by the Research Ethics Commission of China‐Japan Friend Hospital (2020‐21‐K16), who waived informed consent due to its observational nature.

## Supporting information


**Table S1.** Characteristics of immunocompromised patients with acute respiratory failure in different types of ECMO therapy.

## Data Availability

The datasets used and/or analysed during the current study are available from the corresponding author on reasonable request.
