# Supplementary material for: Extracorporeal membrane oxygenation in immunocompromised patients with acute respiratory failure: A retrospective cohort study
Source: Clin Respir J. 2023 Aug 27;17(9):874–83. doi: 10.1111/crj.13674 (PMC10500325; doi:10.1111/crj.13674)
Supplement: Supplementary file 1 — Table S1. Characteristics of immunocompromised patients with acute respiratory failure in different types of ECMO therapy. [file CRJ-17-874-s001.docx]

Supplementary Table 1. Characteristics of immunocompromised patients with acute respiratory failure in different types of ECMO therapy.

| Variables | | Total  (n = 46) | “Awake” ECMO  (n = 19) | | Intubated ECMO  (n = 27) | p |
| --- | --- | --- | --- | --- | --- | --- |
| Gender, n (%) | |  |  | |  | 0.968 |
| Female | | 18 (39.1) | 8 (42.1) | | 10 (37.0) |  |
| Male | | 28 (60.9) | 11 (57.9) | | 17 (63.0) |  |
| Age, Median (IQR) | | 60 (50.0, 66.0) | 63 (57.5, 68.5) | | 56 (42.5, 64) | 0.055 |
| BMI, Mean ± SD | | 24.2 ± 3.0 | 24.8 ± 2.7 | | 23.8 ± 3.1 | 0.279 |
| Type_of_immunosuppression, n (%) |  | | |  | | 0.755 |
| Long-term/high-dose steroids or other  immunosuppressant drugs | | 42 (91.2) | 18 (94.7) | | 24 (88.9) |  |
| Active hematologic malignancy | | 2 (4.4) | 0 (0) | | 2 (7.4) |  |
| Active solid organ malignancy | | 2 (4.4) | 1 (5.3) | | 1 (3.7) |  |
| SOFA, Median (IQR) | | 6 (5, 8) | 6 (5, 7) | | 6 (5, 8) | 0.462 |
| Apache_II, Median (IQR) | | 20 (16.3, 24.0) | 19 (15.0, 22.0) | | 21 (17.0, 26.5) | 0.336 |
| Pre_ECMO PaO_2_/FiO_2_ ratio, Mean ± SD | | 66.5 ± 14.5 | 64.5 ± 11.3 | | 67.9 ± 16.3 | 0.408 |
| Pre_ECMO CD_4_ level, Median (IQR) | | 213 (150.3, 325.3) | 218 (168.0, 361.5) | | 210 (130.0, 313.5) | 0.3 |
| RR decline, Median (IQR) | | 7（3.3，15.0） | 15（6.5，17.5） | | 5（2.0，8.0） | 0.003 |
| Leucocytes count (× 10^9^/L), Mean ± SD | | 13.4 ± 7.0 | 14.0 ± 6.9 | | 13.0 ± 7.2 | 0.65 |
| Lymphocyte count (× 10^9^/L), Median (IQR) | | 0.6 (0.3, 0.7) | 0.6 (0.3, 0.9) | | 0.5 (0.3, 0.6) | 0.16 |
| Platelets (× 10^9^/L), Median (IQR) | | 144.5 (107.0, 220.5) | 196.0 (106.5, 250.0) | | 129.0 (108.0, 183.5) | 0.26 |
| Hemoglobin g/L, Mean ± SD | | 107.2 ± 22.3 | 110.0 ± 20.6 | | 105.3 ± 23.6 | 0.485 |
| Lactate dehydrogenase, Median (IQR) | | 521.5 (387.5, 743.5) | 432.0 (368.5, 524.5) | | 627.0 (422.0, 806.5) | 0.048 |
| C-reactive protein, Median (IQR) | | 8.1 (5.2, 15.2) | 9.2 (6.3, 15.5) | | 7.3 (4.9, 14.0) | 0.349 |

ECMO = extracorporeal membrane oxygenation, IQR= interquartile range, BMI= Body Mass Index, SOFA= sequential organ failure assessment, RR=respiratory rate.
